# The Behavior of the Type of Peritoneal Transport in the Inflammatory and Oxidative Status in Adults Under Peritoneal Dialysis

**DOI:** 10.3389/fmed.2019.00210

**Published:** 2019-09-27

**Authors:** Julio Alejandro Gutiérrez-Prieto, Javier Soto-Vargas, Renato Parra-Michel, Héctor Leonardo Pazarín-Villaseñor, Andrés García-Sánchez, Alejandra Guillermina Miranda-Díaz

**Affiliations:** ^1^Servicio de Nefrología, Hospital General Regional No. 46, Instituto Mexicano del Seguro Social, Guadalajara, Mexico; ^2^Instituto de Terapéutica Experimental y Clínica, Centro Universitario de Ciencias de la Salud, Universidad de Guadalajara, Guadalajara, Mexico

**Keywords:** peritoneal dialysis, peritoneal transport, oxidative stress, oxidative DNA damage, inflammation

## Abstract

Peritoneal dialysis (PD) is an alternative for managing the end-stage renal disease (ESRD). The peritoneal membrane (PM) is not just a membrane that passively responds to diffusion and convection. The characteristics of PM result in the peritoneal equilibrium test (PET) and with this test is possible to obtain the type of peritoneal transport (PT). The patient on PD can be classified in different types of PT as; Low, Low Average, High Average, and High. The aim of the study was to compare the inflammatory cytokines, oxidants, antioxidants, and oxidative DNA damage markers in the different types of PT. A cross-sectional analytical study of 77 adult PD patients was performed. Levels of lipoperoxides (LPO) were higher in all types of PT vs. healthy volunteer controls (HC) (*p* < 0.0001). Nitric oxide (NO) levels were found significantly down-regulated in all types of PT (*p* < 0.0001). The activity of the superoxide dismutase enzyme (SOD) was found to be significantly increased in all types of PT vs. the HC (*p* < 0.0001). The levels of the DNA repair enzyme were found to be decreased in all types of PT. The levels of the pro-inflammatory cytokines TNF-α, IL-6, the marker of oxidative DNA damage, 8-IP and the total antioxidant capacity (TAC) were all significantly decreased, contrary to the levels in HC, possibly by the clearance in the dialysis fluid in all types of PT or due to down-regulation of their expression. In conclusion, we found significant changes in markers of inflammation, oxidative stress, and oxidative damage to DNA in all types of PT; Low, low average, high average, and high PT in the values of D/P creatinine at 4 h compared to HC.

## Introduction

End-stage renal disease (ESRD) is a clinical morbidity given the increasing prevalence of diseases as diabetes mellitus (DM) and hypertension. Mortality in patients with ESRD has decreased 30% since 1999 (1). In Jalisco (Mexico), nearly half of the patients (49.4%) are on PD (2). The adequacy of initial and maintenance PD prescription it is usually based on Twardowski's peritoneal equilibrium test (PET) result and KT/V. PET is used to evaluate the transport function of the peritoneal membrane (PM) of each patient, in which the solute transport rates (creatinine, sodium, urea, glucose) are evaluated by the speed of the equilibrium reached between the dialysate and the peritoneal capillaries. According to the PET result, the patient on PD can be classified in different types of peritoneal transport (PT) as; Low, Low Average, High Average and High (3, 4). Some studies refer to High PT as a risk factor for mortality and technique failure in PD patients (5, 6). However, other authors showed that a high PT by itself is not an independent risk factor for either mortality or technique failure and exposed that other factors, like peritoneal protein clearance, residual renal function and age, are better predictors for survival rather than peritoneal membrane (PM) permeability (7–9). Hence, the effect of PM solute transport rate on these findings remains unclear. Cardiovascular disease (CVD) is the leading cause of death among patients in hemodialysis (HD) or peritoneal dialysis (PD) (10). Inflammation is one of the earliest events in situations of cardiac stress which implies high levels of endothelial adhesion molecules, increased production and release of inflammatory cytokines and chemokines (11). Studies have reported inflammation as an independent risk factor for all causes of cardiovascular mortality in HD and PD patients (12). Oxidative stress (OS) is characterized by an imbalance between oxidants and antioxidants (13). OS is implicated as a unifying factor between chronic kidney disease (CKD) and CVD (14), and it is involved in mechanism related to vascular calcification, oxidation of low-density cholesterol, and low endothelial nitric oxide (NO) which lead to atherosclerosis (15–18). The increase in reactive oxygen species (ROS) and the decrease in antioxidants among PD patients in counterpart with matched controls have been previously reported (19, 20). The marker of oxidative damage to DNA; 8-hydroxy-2-deoxyguanosine (8-OHdG) is considered as an independent predictor of all-cause mortality in patients undergoing dialysis (21). An increase of OS may lead to increased risk for cardiovascular disease in patients in DP regardless of the type of PT, however, there is no clear association, whereas OS is clearly associated with PM permeability rate.

The aim of the study was to compare the serum/plasma levels of inflammatory cytokines, oxidants, antioxidants, and oxidative DNA damage markers in the different types of PT.

## Patients and Methods

An analytical cross-sectional study was conducted. Seventy-seven ESRD patients receiving PD at Hospital General Regional No. 46 of the Instituto Mexicano del Seguro Social in Jalisco, Mexico were included. Patients were seen in the PD outpatient clinic and were sent to perform the PET. Patients were eligible of the PD program and never had have a PET performed before. Patients with episodes of peritonitis in the last 6 months with dysfunction of the PD catheter, with a current infectious, inflammatory, or malignant process were excluded.

### Data Collection

Demographic and clinical data were obtained through medical records the day that PET was performed. An extra venous blood sample during PET was obtained to assess the following: hemoglobin, white blood cell count, blood urea nitrogen, creatinine, glucose, albumin, sodium, potassium, chloride, calcium, phosphorus, uric acid, total cholesterol, high-density lipoprotein cholesterol, low-density lipoprotein cholesterol, very low-density cholesterol, triglyceride, bicarbonate, serum iron, and transferrin saturation.

### Peritoneal Equilibrium Test

The PET was performed and the patients were classified based on the result of dialysate to plasma/creatinine ratio at 4 h as Low, Low Average, High Average, and High PT. The PET was performed in 149 patients a minimum of sixteen patients were collected in each group of type of PT. For the calculation of sample size, we used the formula to compare two means. We assumed a risk of making two tail type I and type II errors of 5–20%, respectively. Previous data of OS markers measured in this study were not available in the populations of Low, Low Average, High Average, and High PT. Thus, we took as reference the variation of NO between PD patients and HC as reported by Uzun et al. (22). An additional 20% sample was added for possible withdrawals, missing data, loss of sample while processing, etc.

For the measurement of metabolites, blood samples were drawn when PET blood samples were taken and centrifuged at 10,000 rpm for 10 min at room temperature; supernatants were stored in aliquots at −80°C until final processing. We included 10 mL of extra blood from 10 blood donors [healthy volunteer controls (HC)] to establish the normal value of the metabolites. For the selection of blood donors, we considered subjects who are not known to suffer any relevant illness to the proposed study and be within normal range of body measurements (23). Also, efficient measures were implemented to improve the sample result among them; a better examination of donors through microbiological tests has led to the reduction of transfusion-related infections (1 in 8 million for HIV, 1 in 6.7 million hepatitis C viruses and 1 in 1.7 million for hepatitis B viruses) (24).

### Inflammation Cytokines

#### TNF-α and IL6

The ELISA kit was used to determine the IL-6 and TNF-α level. We followed the instructions of the kit manufacturer (Peprotech, Rocky Hill, NJ 08553, USA^®^). Both cytokines had a detection limit of 32 pg/mL. First, 100 μL of diluted capture antibody was added, followed by incubation overnight at room temperature. Then, 300 μL of blocking buffer was added to the wells and it was incubated for 1 h at room temperature. Plasma and standards were added, followed by incubation for 2 h at room temperature. After several washings, 100 μL of diluted detection antibody was added. The plate was incubated at room temperature for 2 h. Then, 100 μL diluted HRP-Avidin-conjugate was added, followed by incubation for 30 min at room temperature. Finally, 100 μL of substrate solution was added to each well. The plate was read at a wavelength of 405 nm with correction set at 650 nm. The TNF-α intra-assay coefficient of variation was 2.1% and the intra-assay IL-6 was 4.7%. For all of the technical readings of optical density the Synergy HT (BIOTEK) microplate reader was used.

### Oxidative Stress Markers

#### Products of Lipid Peroxidation

The serum levels of LPO were measured through the FR22 assay kit (Oxford Biomedical Research Inc., Oxford, MI, USA^®^) according to the manufacturer's instructions. The limit of detection for this test was 0.1 nmol/mL. The chromogenic reagent reacts with malondialdehyde (MDA) and 4-hydroxy-alkenals to form a stable chromophore. One hundred and forty microliter of plasma with 455 μL of N-methyl-2-phenylindole in acetonitrile (Reagent 1) was diluted with ferric iron in methanol. Samples were agitated, after 105 μL 37%, HCl was added, followed by incubation at 45°C for 60 min, and centrifugation at 12,791 rpm for 10 min. One hundred and fifty microliter of the supernatant was added to a microplate and absorbance was measured at 586 nm. The standard curve with known concentrations of 1,1,3,3-Tetramethoxypropane in Tris-HCl was used. The intra-assay CV was 8.5%.

#### 8-Isoprostane

The immunoassay reagent kit from Cayman Chemical Company^®^ (Michigan, USA) was used according to the manufacturer's instructions. The limit of detection was 0.8 pg/mL. The 8-IP assay was based on the principle of competitive binding between sample 8-IP, 8-IP acetyl cholinesterase (AChE) conjugate, and 8-IP tracer. Fifty microliter of serum samples or standard were added to each well and 50 μL of 8-IP AChE tracer was added to all wells except the total activity and blank wells. Fifty microliter of 8-IP enzyme immunoassay antiserum was added to all wells except the total activity and blank wells. Fifty microliter of 8-IP antiserum was added to all wells except total activity, non-specific binding, and blank wells. The plate was covered and incubated at 4°C for 18 h and then washed 5 times with buffer. Absorbance was read at 420 nm. The intra-assay CV was 12.5%.

#### Nitric Oxide

The serum samples were de-proteinized by the addition of zinc sulfate; 6 mg of zinc sulfate was added to 400 μL of sample, vortexed for one min and the samples were centrifuged at 10,000 × g for 10 min at 4°C. For the determination of NO, the colorimetric method was used according to the kit (Nitric Oxide Assay Kit, User protocol 482650, Calbiochem^®^). Eighty five microliter of the standard or sample were added to the wells of the plate, 10 μL of nitrate reductase was added to each well and 10 μL of 2 mM NADH. The plate was shaken for 20 min at room temperature. Fifty microliter of dye 1 was added and shaken briefly; 50 μL of dye 2 was added and again shaken for 5 min at room temperature. Finally, the plate was read at 540 nm in a spectrophotometer within the first 20 min after the procedure was finished.

### Antioxidants

#### Superoxide Dismutase (SOD)

The kit manufacturer's instructions were followed (SOD No. 706002, Cayman Chemical Company^®^, USA) for the detection of ${\text{O}}_{2}^{-}$ generated by xanthine oxidase and hypoxanthine enzymes through the reaction of tetrazolium salts. The serum samples were diluted 1:5 in sample buffer, 200 μL of the radicals' detector diluted 1:400, was placed, and 10 μL of the sample was added. After slow agitation, 20 μL of xanthine oxidase was added to the wells. The microplate was incubated for 20 min at room temperature and the absorbency was read at a wavelength of 440 nm. The levels are reported in U/mL.

#### Total Antioxidant Capacity

The total antioxidant capacity (TAC) evaluation were made following the instructions of the kit manufacturer (Total Antioxidant Power Kit, No. TA02.090130, Oxford Biomedical Research^®^), to obtain the concentration in mM equivalents of trolox (an analog of vitamin E). The detection limit was 0.075 mM. The serum samples and standards were diluted 1:40, and 200 μL were placed in each well. The plate was read at 450 nm as a reference value, 50 μL of copper solution was added, and the plate was incubated at room temperature for 3 min. Fifty microliter of stop solution was added and the plate was read again at 450 nm. The dilution factor was considered in the result. The intra-assay CV was 7.8%.

### 8-hydroxy-2′-deoxyguanosine (8-OHdG)

The method by the manufacturer of the ELISA kit was followed (8-hydroxy-2′-deoxyguanosine No. ab201734 Abcam^®^, Cambridge, United Kingdom). An 8-OHdG-coated 96-well plate was used. Fifty μL of sample or standard were added to each well and were detected with horseradish peroxidase (HRP) conjugated 8-OHdG antibody. Tetramethylbenzidine (TMB) substrate was used to obtain a color signal measured at 450 nm using a spectrophotometer reader. The value of the samples was plotted on the standard curve to interpolate sample concentrations.

### 8-oxoguanine-DNA-N-glycosylase-1 (hOGG1)

Repair of the oxidative damage of DNA was determined with a commercial kit (hOGG1 MBS702793 My BioSource^®^, San Diego, CA). The manufacturer's instructions were followed. One hundred microliter of plasma and standards were added to the wells and the plate was incubated at 37°C. The biotinylated antibody was added and incubated under the same conditions. The corresponding washings were done and the HRP-avidin was added, followed by the substrate and the stop solution at the corresponding times. The optical density was read at 450 nm.

### Statistical Analysis

All data were expressed as mean ± standard deviation (SD), standard error (SEM), or percentages. Kolmogorov-Smirnov test was used to analyze distribution normality of measured parameters. Differences between the four groups for continuous variables we used Kruskall-Wallis or a way ANOVA test according to the normality distribution. The *post-hoc* Dunn-Bonferroni correction was used for pairwise comparison and was considered significant at *p* ≤ 0.05. For categorical variables, the difference between the groups using *Chi*^2^ or Fisher's exact test for categorical variables. To evaluate relationships between oxidative stress markers and PET results Pearson's correlation test was used. *p* ≤ 0.05 was considered statistically significant. SPSS for Windows version 18.0 (IBM SPSS statics Inc., Chicago, IL, USA) was used.

### Ethical Considerations

The research complies with the ethical principles for medical research in human beings stipulated in the Declaration of Helsinki 64th General Assembly, Fortaleza, Brazil, October 2013. To adhering to the standards of Good Clinical Practices. All procedures were performed according to regulations stipulated in the General Health Legal Guidelines for Health care Research in Mexico, 2nd Title, in Ethical Aspects for Research in Human Beings, Chapter 1, and Article 17. All patients gave and signed informed consent form in the presence of signed witnesses. The study was evaluated and approved by the Local Ethics, and Research Committee, at the Hospital General Regional No. 110, Instituto Mexicano del Seguro Social (R-2017-1303-117).

## Results

### Baseline Demographic and Clinical Characteristics

Of 77 patients who met the inclusion criteria; 15 were Low (It was not possible to complete 16 patients of this group), 23 Low Average, 23 High Average, and 16 were classified as High PT. The median age was 42 (IQR 27–60) years, 53 (65%) patients were male, and the etiology of CKD was of unknown and DM kidney disease predominantly. The average time on PD program was ~40 months. Albumin (*p* = 0.05) and Triglycerides levels (*p* = 0.007) were lower in the High PT compared to the rest of the transporters. The PET was significant in the High PT patients (*p* < 0.0001*)*. Hypertension was found more frequently in the High Average group (*p* = 0.016). Uric acid increased in patients with Low/Low Average PT rates compared with those with High Average/High PT rates (*p* = 0.016). Baseline demographic and characteristics of PD therapy according to the type of PT are shown in Tables 1, 2.

**Table 1:** Clinical data of different type of peritoneal transport.

	**Low n-15**	**Low average n-23**	**High average n-23**	**High n-16**	***p***
**Antihypertensive therapy** *n* (%)					
Enalapril	0	2 (8.7)	2 (8.7)	1(6.2)	0.57
Losartan	12 (80)	17 (73.9)	15 (65.2)	11 (68.8)	0.001^*^
No treated	3 (20)	4 (17.4)	6 (26.1)	4 (25)	0.049^*^
**Dialysis dose** *n* (%)					
2,000 mL 1.5% every 6 h	6 (39)	11 (47.8)	9 (39.1)	5 (31.2)	0.002^*^
2,000 mL 1.5% and 2.25% every 6 h alternating	7 (46.7)	6 (26.1)	8 (34.8)	7 (43.8)	0.13
2,000 mL 2.25% every 6 h	2 (13.3)	6 (26.1)	6 (26.1)	4 (25)	0.049^*^
**Type of PD** *n* (%)					
APD	4 (26.7)	3 (13)	5 (21.7)	3 (18.8)	0.35
CAPD	11 (73.3)	20 (87)	18 (78.3)	13 (81.2)	<0.001^*^
**CKD etiology** *n* (%)					
Unknown	5 (33.3)	11 (47.8)	12 (52.2)	8 (50)	0.004^*^
Nephrolithiasis	1 (6.7)	0	0	0	1.000
Diabetes mellitus	7 (46.6)	12 (52.2)	8 (34.8)	7 (43.8)	0.002^*^
Preeclampsia with CKD	0	0	2 (8.7)	0	0.56
Polycystic kidney disease	1 (6.7)	0	0	0	1.00
Hypertension	1 (6.7)	0	1 (4.3)	1 (6.2)	0.67

*CKD, Chronic Kidney Disease, Values are media ± SD or percent; PD, peritoneal dialysis; APD, ambulatory peritoneal dialysis; CAPD, continuous ambulatory peritoneal dialysis*.

* *Chi^2^ test*.

**Table 2:** Demographic and biochemical data according to the type of peritoneal transport.

	**Low n-15**	**Low average n-23**	**High average n-23**	**High n-16**	***p***
**Gender** ***n*** **(%)**					
Male	11 (73.33)	16 (69.56)	18 (78.26)	8 (50)	<0.000^= t^
Female	4 (26.67)	7 (30.44)	5 (21.74)	8 (50)	0.475
Age (years)	47.33 ± 16.97	44.45 ± 18.62	43.88 ± 19.66	46.59 ± 17.60	0.894
Weight (kg)	67.06 ± 14.15	72.12 ± 13.41	68.63 ± 11.25	71.68 ± 10.68	0.408
Height (m)	1.63 ± 0.07	1.64 ± 0.09	1.66 ± 0.08	1.61 ± 0.11	0.163
BMI kg/m^2^	25.37 ± 5.37	26.70 ± 4.3	24.86 ± 3.61	28.13 ± 6.32	0.067
Hypertension *n* (%)	6 (40.00)	10 (43.06)	14 (60.29)	8 (51.39)	0.016^= t^
Residual urine (mL)	813.89 ± 566.93	655.26 ± 587.34	697.00 ± 579.32	582.35 ± 451.71	0.687
PET	0.55 ± 0.04^a^	0.66 ± 0.03^b^	0.78 ± 0.03^c^	0.90 ± 0.04^d^	<0.000^**^
Hemoglobin (g/dL)	12.04 ± 1.95	11.13 ± 2.17	11.02 ± 2.07	10.22 ± 1.99	0.086
Urea (mg/dL)	113.22 ± 26.10	116.95 ± 30.09	107.51 ± 37.16	125.73 ± 32.38	0.254
Creatinine (mg/dL)	10.44 ± 5.06	10.75 ± 4.54	11.52 ± 4.83	11.49 ± 4.88	0.809
Glucose (mg/dL)	137.98 ± 88.64	124.71 ± 59.76	114.25 ± 43.53	102.90 ± 33.72	0.195
Phosphorus (mg/dL)	5.05 ± 1.68	4.85 ± 1.73	4.66 ± 1.88	5.62 ± 1.44	0.284
Calcium (mg/dL)	9.40 ± 0.91	9.52 ± 1.16	9.22 ± 1.07	9.28 ± 0.99	0.422
Magnesium (mg/dL)	2.23 ± 0.47	2.21 ± 0.37	2.26 ± 0.53	2.24 ± 0.46	0.976
Potassium (mEq/L)	4.49 ± 0.60	4.32 ± 0.63	4.23 ± 0.71	4.49 ± 0.70	0.437
Sodium (mEq/L)	139.47 ± 3.43	138.86 ± 3.00	138.68 ± 3.82	136.64 ±3.82	0.159
Albumin (g/dL)	3.80 ± 0.51	3.58 ± 0.67	3.40 ± 0.70	3.25 ± 0.46	0.053
Uric acid (mg/dL)	6.85 ± 1.65^a, b^	6.92 ± 1.55^a^	5.99 ± 0.86^b^	6.13 ± 0.91^a, b^	0.016^**^
Iron (mcg/dL)	92.91 ± 45.15	65.27 ± 28.08	76.05 ± 28.87	80.71 ± 44.22	0.063
TSAT %	41.25 ± 7.93	28.7 ± 23.77	40.56 ± 23.22	18.67 ± 0.58	0.330
Cholesterol (mg/dL)	193.24 ± 46.74	166.42 ± 50.68	161.89 ± 42.5	179.51 ± 44.27	0.100
HDL (mg/dL)	37.11 ± 9.17	40.51 ± 13.43	45.88 ± 15.20	50.46 ± 17.0	0.033^**^
LDL (mg/dL)	112.93 ± 30.56	93.04 ± 41.12	93.43 ± 35.25	96.58 ± 30.00	0.333
Triglycerides (mg/dL)	226.69 ± 159.22^a^	158.61 ± 87.03^a, b^	127.53 ± 68.38^b^	129.99 ±74.92^b^	0.007^*^
VLDL (mg/dL)	45.71 ± 32.63^a^	32.93 ± 19.08^a, b^	25.44 ± 13.71^b^	26.06 ±15.05^a, b^	0.010^*^
HCO_3_ (mEq/L)	20.19 ± 2.93	21.01 ± 3.21	21.47 ± 3.32	20.37 ± 3.81	0.574
CRP (mg/L)	4.293 ± 3.29	8.78 ± 10.90	3.58 ± 4.84	5.12 ± 5.81	0.113

Values are mean ± SD or percent. PET, peritoneal equilibration tests; BMI, body mass index; TSAT, transferrin saturation; HDL, high-density lipoprotein; LDL, low-density lipoprotein; VLDL, very low-density lipoprotein; HCO_3_, bicarbonate; CRP, C reactive protein; = t Chi^2^ test;

* K-W, Kruskall Wallis test;

** *ANOVA. a,b,c,d: different letter denote statistically significant difference using Dunn-Bonferroni pairwise test at p ≤ 0.05*.

### Inflammatory Cytokines According the Type of PT

#### TNF-α and IL-6

The TNF-α level in the different types of PT were found to be significantly decreased. Low PT, 28.94 ± 5.23 pg/mL, Low Average, 27.90 ± 4.20 pg/mL, High Average, 32.56 ± 6.18 pg/mL and High, 66.28 ± 21.12 pg/mL vs. levels found in HC, 631.38 ± 125.40 pg/mL (*p* < 0.0001 Mann Whitney *U*-test and Kruskall-Wallis test).

The different types of PT showed lower serum levels of IL-6; Low PT, 326.13 ± 287.75 pg/mL, Low Average, 124.89 ± 47.25 pg/mL, High Average 357.21 ± 126.47 pg/mL and High PT, 272.02 ± 164.65 pg/mL vs. serum levels of IL-6 in HC, 498.45 ± 93.96 pg/mL (*p* < 0.0001 Mann Whitney *U*-test and Kruskall-Wallis test) (Table 3).

**Table 3:** Inflammation cytokines, oxidative DNA damage, oxidants and antioxidants according to the type of peritoneal transportation of patients in peritoneal dialysis.

	**Healthy controls n-10**	**Low n-15**	**Low average n-23**	**High average n-23**	**High n-16**	***p* K-W**
**Inflammatory cytokines**						
TNF-α (pg/mL)	631.38 ± 125.40^b^	28.94 ± 5.23^a^	27.90 ± 4.20^a^	32.56 ± 6.18^a^	66.28 ± 21.12^a^	<0.0001
IL-6 (pg/mL)	498.45 ± 93.96^b^	326.13 ± 287.75^a^	124.89 ± 47.25^a^	357.21 ± 126.47^a^	272.02 ± 164.65^a, b^	0.001
**Oxidative DNA damage**						
8-OHdG (ng/mL)	65.21 ± 19.14^b^	38.25 ± 15.10^a, b^	38.09 ± 20.68^a, b^	11.20 ± 3.05^a^	31.05 ± 8.02^a^	0.046
hOGG1 (ng/mL)	3.53 ± 2.09	0.29 ± 0.17	0.30 ± 0.12	0.07 ± 0.01	0.23 ± 0.09	0.38
**Oxidants**						
8-Isoprostanes (pg/mL)	22.88 ± 3.80^b^	19.05 ± 15.83^a^	4.76 ± 0.79^a^	3.54 ± 0.42^a^	6.06 ± 1.46^a^	<0.0001
Lipoperoxides (μM)	3.05 ± 0.18^b^	160.01 ± 30.39^a^	157.83 ± 35.48^a^	223.77 ± 90.26^a^	101.80 ± 21.94^a^	<0.0001
Nitric oxide (μM)	197.97 ± 34.20^b^	20.13 ± 4^a^	23.53 ± 5.35^a^	6.82 ± 1.44^a^	10.59 ± 3.52^a^	<0.0001
**Antioxidants**						
Superoxide dismutase (U/mL)	0.23 ± 0.02^b^	3.02 ± 0.38^a^	3.40 ± 0.37^a^	3.36 ± 0.35^a^	3.41 ± 0.22^a^	<0.0001
Total antioxidant capacity (mM)	2.62 ± 0.17^b^	0.71 ± 0.05^a^	0.68 ± 0.02^a^	0.64 ± 0.03^a^	0.68 ± 0.05^a^	<0.0001

*Values are media ± SEM, K-W, Kruskall Wallis test. a,b: different letter denote statistically significant difference using Dunn-Bonferroni pairwise test at p ≤ 0.05*.

### Oxidative Damage of DNA and Repair

Levels of the oxidative DNA damage marker were significantly reduced in Low PT, 38.25 ± 15.10 ng/mL, Low Average, 38.09 ± 20.68 ng/mL (*p* = 0.042), High Average, 11.20 ± 3.05 ng/mL, and High PT 31.05 ± 8.02 ng/mL (*p* = 0.046) in contrast to HC, 65.21 ± 19.14 ng/mL (*p* = 0.004).

The hOGG1 levels in HC obtained 3.53 ± 2.09 ng/mL. The different types of PT obtained lower levels (NS); Low PT, 0.29 ± 0.17 ng/mL, Low Average, 0.30 ± 0.12 ng/mL, High Average, 0.07 ± 0.01 ng/mL, and High 0.23 ± 0.09 ng/mL (Table 3).

### Oxidants

The serum levels of the 8-IP were significantly reduced (*p* < 0.0001) in all types of PT in relation to the levels of HC, 22.88 ± 3.80 pg/mL. The serum levels of LPO in the HC were 3.05 ± 0.18 μM, however, significantly increased levels of LPO were found (*p* < 0.0001) in Low PT, 160.01 ± 30.39 μM, Low Average, 157.83 ± 35.48 μM, High Average, 223.77 ± 90.26 μM, and High PT, 101.80 ± 21.94 μM.

In contrast, the levels of NO metabolites were found significantly decreased in the different types of PT; Low 20.13 ± 4.79 μM, Low Average 23.53 ± 5.35 μM, High Average 6.82 ± 1.44, and High 10.59 ± 3.52 μM vs. levels of HC, 197.97 ± 34.20 μM (*p* < 0.0001) (Table 3).

### Antioxidants

Serum activity of SOD was found increased in all types of PT; Low 3.02 ± 0.38 U/mL, Low Average, 3.40 ± 0.37 U/mL, High Average, 3.36 ± 0.35 U/mL, and High 3.41 ± 0.22 U/mL. In contrast, the activity of the enzyme in HC obtained 0.23 ± 0.02 U/mL (*p* < 0.0001) (Table 3).

The levels of TAC were found to be significantly consumed in all types of PT; Low 0.71 ± 0.05 mM, Low Average 0.68 ± 0.02 mM, High Average 0.64 ± 0.03 mM, and High PT, 0.68 ± 0.05 mM vs. the HC, 2.62 ± 0.17 mM (*p* < 0.0001) (Table 3).

### Correlations

Table 4 shows correlations found between the markers of inflammation and oxidative stress with PET values. Only NO presented a low negative correlation with PET (*r* = −0.269, *p* = 0.021).

**Table 4:** Pearson correlation coefficient between the oxidative stress markers and PET value.

	***r***	***p***
8-OH-dG ng/mL	−0.040	0.735
hOGG1 ng/mL	−0.053	0.642
LPO μM	−0.034	0.766
Nitric oxide μM	−0.269*	0.021
8-isoprostane pg/mL	−0.175	0.129
SOD U/mL	0.086	0.447
Total antioxidant capacity mM	−0.090	0.429
TNF-α pg/mL	0.221	0.055
IL-6 ng/mL	0.048	0.682
SOD U/mL	0.086	0.447

## Discussion

In this study, we investigated the behavior between the types of PT and the levels of diverse markers of inflammation and OS in patients with ESRD on PD. The levels of the above-mentioned markers were altered in the totality of the 77 patients in comparison with the levels of these markers in HC. Uremic inflammation commonly affects patients with CKD and is characterized by increased expression of pro-inflammatory cytokines (25, 26). Overexpression of TNF-α and IL-6 has been previously reported in patients with ESRD (27). Systemic pro-inflammatory cytokines is a significant risk factor suggestive of infection in patients with PD (28). However, recently it was reported that circulating pro-inflammatory cytokines TNF-α and IL-6 decreased significantly after 3 months of PD and remained low at 6 months of follow-up in patients with heart failure undergoing PD due to overflow (29). The patients included in the study did not have heart failure, but the information could partially explain the behavior of the pro-inflammatory cytokines in the included patients. According to the types of PT, the serum levels of TNFα and IL-6 were significantly reduced. In this finding, we can hypothesize that the systemic inflammatory state was not present or that in the PD process the cytokines were purified in the dialysis fluid or is the result of a change in its expression (TNF-α and IL-6 in the dialysis fluid were not determined).

The circulating levels of 8-OHdG were first described in 1984 (30). 8-OHdG is considered a robust and sensitive marker of the OS; it reflects the oxidative decomposition of DNA (18). The 8-OHdG marker is considered an independent predictor of all-cause mortality in HD patients (21). Overexpression of 8-OHdG produces erroneous readings by converting A to C and form G to T capable of producing mutagenic alterations (31). Circulating and urinary concentrations of the 8-OHdG marker are increased in patients with chronic diseases such as cancer, chronic hepatitis, DM, CVD, and ESRD (32–34). In a study reported in 2015, the authors analyzed the levels of 83 PD patients and found higher levels of 8-OHdG than the control population without significant difference between the types of PD. In other reports, the higher levels were found in patients on HD, which underscores the importance of the role of other factors involved in oxidative status (19). Contrary to previous reports, we found significantly decreased levels of the 8-OHdG marker in Low Average PT, High Average and High PT in contrast to levels obtained in HC. Our findings could be due to the possible depuration of this marker during PD sessions due to greater biocompatibility of the PD solutions in the PM (19). Other studies have shown moderate positive correlation between the ratio of creatinine D/P at 4 h and the levels of 8-OHdG in the peritoneal effluent as a marker of damage to the PM. However, the authors did not measure plasma levels and did not explain whether the correlation is due to increased local production of 8-OHdG or to a better clearance of it (35). The clearance of this marker in the dialyzed effluent is only speculative. We did not find in the available literature low systemic levels of 8-OHdG as in our study.

In the present study, the levels of the DNA repair enzyme (hOGG-1) were found to be decreased in all types of PT (NS), in contrast to levels obtained in HC, suggesting the inactivity or consumption of the enzyme in an attempt to avoid over-production of the 8-OHdG. We can speculate that the results may be due to the inactivity of the enzyme or down-regulation of its expression. The repair of DNA containing oxidized bases, involves complex pathways (36). One mechanism of repair that eliminates 8-OHdG is characterized by the cleavage of the glycosidic bond of the 8-OHdG residue by breaking the phosphodiester bond at the resulting apurinic site by β-elimination (37). There is a lack of data in the literature about hOGG-1 in dialysis patients. We previously evaluated the long-term behavior of hOGG-1 in patients with renal transplantation and we found low levels of hOGG-1 in HC compared with patients in HD or DP before transplantation. However, these measures were made with no distinction of type of PT or renal replacement therapy (38).

LPO are molecules that result from the reactions of ROS (39). The production of ROS by mitochondria is important because it underlies oxidative damage in many pathologies and contributes to retrograde redox signaling from the matrix of mitochondria to the cytosol and nucleus (40). MDA is a final compound of the LPO process and is useful as an indicator of OS in patients with CKD where its elimination is decelerated (41). The lipid peroxidation process is involved in the genesis of atherosclerotic lesions and cardiovascular complications in CKD (42–44). Higher levels of MDA have been reported in patients with ESRD, HD, PD vs. HC (45, 46). There are also reports where no differences in MDA levels in patients with PD and HC (47). In our study, we found increased levels of LPO in all types of PT (48) and no correlation with the value of PET. Stepanova et al. previously reported the association of MDA with some PD parameters, they also found no correlation with D/P creatinine ratio but a negatively correlation with total KT/V (49).

NO is produced by an NOS family and consists of three identified isoforms (neuronal, inducible, and endothelial NOS). NO is an important molecule in physiological processes as in neuronal transmission, reproduction, apoptosis and predominantly in endothelial vasodilatation. NO also actively participates in pathological processes such as inflammation, septic shock, asthma, and nitrosative stress. NOS produces ${\text{O}}_{2}^{-}$ by NOS decoupling phenomenon (50). The generation of ${\text{O}}_{2}^{-}$ occurs mainly when the NOS is not coupled with its cofactor or substrate due to depletion of L-arginine (10). Vascular endothelial dysfunction is due to the decoupling of NOS, phagocytic activation of vascular membrane oxidases and mitochondrial. NO is capable of increasing vascular stiffness and compromising cardiac output (51). The decrease in OS is directly related to endothelial dysfunction by reducing the bioavailability of NO to produce alterations in vascular permeability and favoring the entry of low-density cholesterol into the vascular intima where it is oxidized. In this process, the migration of inflammatory cells in the sub-endothelial space takes place after the expression of adhesion molecules by the dysfunctional endothelium (52). In our study, NO levels were significantly decreased in all types of PT vs. the levels found in HC. Additionally, a negative correlation between NO and PET was found. These explained that patient with High and High Average PT tents to have lower levels of NO, which could suggest alterations in the bioavailability of NO in the vascular endothelium (53). Schmidt et al., showed that patients in PD have low levels of NO vs. HC when both are undergoing a controlled dietary NO intake (54). Uzun et al., also found that patients in PD, without HD, have lower levels of NO compared with HC (22). However, Kovačević et al., found increased levels of NO in CAPD (55). The reason of these different outcomes may be explained by the effect of PM solute transport rate among the patient in PD.

8-IP are a bioactive product of peroxidation of arachidonic acid (56). In our study, significantly decreased serum levels were found in the patients studied in comparison to HC. However, in the available literature, one study reported decreased plasma levels of F2-isoprostans in patients with CAPD compared to patients in HD (57). It has been reported down-regulation of this metabolite in people with high consumption of polyunsaturated fatty acids via NOX (58). The enhanced levels of 8-IP has been reported in patients with ESRD receiving HD and CAPD (59). Other studies have found higher levels of 8-IP in CAPD patients vs. healthy matches (60). Currently, there is no defined behavior of 8-IP and its serum levels, as well as the clearance of these in the PD effluent. It would be necessary to determine the levels of this metabolite in the dialytic effluent.

As a defense mechanism against oxidative damage, the organism possesses compounds with antioxidant capacity such as the SOD enzyme. The SOD is a metallo-enzyme that catalyzes the dismutation of ${\text{O}}_{2}^{-}$, this enzyme is considered crucial in the beginning of the enzymatic mechanisms of defense against OS (61). The determination of SOD levels turns out to be an important indicator for the analysis of the OS in various pathologies such as DM and CKD. The activity of SOD enzyme in all types of PT was significantly increased compared to HC in agreement with other studies demonstrating the alteration the OS balance favoring the pro-oxidant state (43). The SOD enzyme contains selenium that is traditionally been considered as the main mediator of antioxidant enzymatic protection (61). In the present study, we found greater activity of the SOD enzyme in all types of PT vs. HC, possibly in an attempt to compensate the increase in LPO products (62, 63). Some studies suggest patients in PD have an increased activity of SOD vs. HC (64).

The main antioxidant defenses in extracellular fluids against OS are bilirubin, uric acid, and albumin; which makes it possible to measure them as a whole by determining the TAC reagent. Albumin, bilirubin and urates prevent free radicals from reacting by sequestering transition metal ions (65–67). We found TAC decreased in all types of PT by contrast to HC, which is consistent with other study that showed low TAC in PD also compared to HC. Other studies demonstrated contrary; higher TAC in patients in PD vs. HC (68). This finding could suggest that these extracellular antioxidants are possibly being cleared in the dialysate effluent.

As strengths, we point out that the present study is one of the largest studies carried out so far at an international level in the PD population with an adequate number of patients in each group according to the type of PT, in addition to being a homogeneous sample as to the baseline, clinical, and therapeutic characteristics. Most of the data available in the literature is focus between binominals of mixed population of Low-Low Average PT and High Average-High PT. In our study, we aimed to show the behavior for each individual group. These data give tools to understand the behavior of the oxidative status in relation of the PM permeability rate, which may contribute to the design of new studies with therapeutic or risk assessment approaches.

In terms of limitations of the study, we accept that it is a cross-sectional study; thus, only one sample was used to decide about the inflammation and OS markers. The levels of the markers do not have a static behavior and a follow up throughout study with multiple determinations in time is required to corroborate these findings.

In conclusion, we found statistically significant changes in the markers of OS, DNA damage, and inflammation in all type of PT, Low, Low Average, High Average, and High PT compared to HC. All patients included had increased levels of LPO and the SOD suggesting compensatory antioxidant activity. Important down-regulation of serum levels of NO with the ability to influence the bioavailability of NO in the renal and systemic vascular endothelium. The decreased levels in hOGG1 was possible due to the down-regulation of the expression or excretion in the dialysis fluid.

## Data Availability Statement

The datasets generated for this study are available on request to the corresponding author.

## Ethics Statement

The study was evaluated and approved by the Local Ethics and Research Committee, at the Hospital General Regional No. 110, IMSS (R-2017-1303-117). The patients/participants provided their written informed consent to participate in this study.

## Author Contributions

JG-P, HP-V, and AM-D: conception and design. AG-S, RP-M, HP-V, and JS-V: analysis and interpretation of data. JG-P, HP-V, AM-D, AG-S, RP-M, and JS-V: document writing and critical review.

### Conflict of Interest

The authors declare that the research was conducted in the absence of any commercial or financial relationships that could be construed as a potential conflict of interest.

## Abbreviations

ESRD: End-stage renal disease
DM: Diabetes mellitus
CVD: Cardiovascular disease
HD: Hemodialysis
PD: Peritoneal dialysis
OS: Oxidative stress
CKD: Chronic kidney disease
ROS: Reactive oxygen species
8-OHdG: 8-hydroxy-2′-deoxyguanosine
DNA: Deoxyribonucleic acid
PET: Peritoneal equilibrium test
KT/V: K, elimination of urea and other waste, from the dialyzer. The t time, the period of each treatment, V is the volume of fluid in the body
PT: Peritoneal transport
PM: Peritoneal membrane
D/P: Transport across the PM for a given solute
HC: Healthy volunteer control
IL-6: Interleukin 6
TNF-α: Tumor necrosis factor alpha
LPO: Products of Lipid peroxidation
MDA: Malondialdehyde
8-IP: 8-Isoprostane
AChE: Acetyl cholinesterase
NO: Nitric Oxide
SOD: Superoxide Dismutase
${\text{O}}_{2}^{-}$: Radical superoxide
TAC: Total antioxidant capacity
HRP: Horseradish peroxidase
TMB: Tetramethylbenzidine
SOD: Superoxide dismutase
HDL: Cholesterol of high density
CAPD: Continuous ambulatory peritoneal dialysis
APD: Ambulatory peritoneal dialysis
BMI: Body mass index
TSAT: Transferrin saturation
LDL: Cholesterol of low-density
VLDL: Cholesterol of very low-density
HCI_3_: Bicarbonate
CRP: C reactive protein.
